# Group B *Streptococcus* among Pregnant Women and Neonates in Saudi Arabia: A Systemic Review

**DOI:** 10.3390/pathogens11091029

**Published:** 2022-09-10

**Authors:** Amer Alshengeti

**Affiliations:** 1 Department of Pediatrics, College of Medicine, Taibah University, Al-Madinah 42353, Saudi Arabia; aalshengeti@dal.ca; 2 Department of Infection Prevention and Control, Prince Mohammad Bin Abdulaziz Hospital, Ministry of National Guard health Affairs, Al-Madinah 42324, Saudi Arabia

**Keywords:** Group B *Streptococcus*, neonates, pregnant women, Saudi Arabia, review

## Abstract

Sepsis caused by Group B *Streptococcus* (GBS) continues to cause mortality and morbidity in newborns, especially in developing countries. Bacterial sepsis in newborns varies nationally and even within countries. Developing countries have reported 34 deaths per 1000 live births compared to 5 in developed countries. This systemic review aimed to assess the prevalence of GBS colonization among pregnant women and the incidence of neonatal GBS sepsis in Saudi Arabia. A literature search of PubMed, MEDLINE Ovid, and Google Scholar was conducted. A total of 21 studies were found: 15 described maternal GBS colonization and 6 studies described neonatal GBS infections. The GBS colonization prevalence among pregnant women ranged from 2.1% to 32.8%. Inconsistencies in the reporting method for neonatal GBS infection rates were observed. Only two studies have the incidence of neonatal GBS as the primary outcome. No national multicenter studies exist on the GBS rates among neonates. Nationwide studies are warranted to assess the burden of GBS infections in neonates. These studies would guide appropriate GBS screening strategies during pregnancy for application in a national public health program.

## 1. Introduction

Despite the advancements in neonatal care, sepsis remains a prominent cause of mortality and morbidity among neonates, particularly in developing countries [1]. Any systemic bacterial infection with a positive blood culture resulting in the first month of life is considered neonatal sepsis [2]. Neonatal infection that is caused by group B *streptococcus* (GBS) bacterium, also known as *Streptococcus agalactiae*, can be classified as early onset and late- onset. The early-onset infection occurs from birth to day 6 of life. The late-onset infection typically occurs from day 7 to day 28 of life (range, 7 through 89 days) [3]. The transmission rate of GBS to newborns from colonized women is approximately 50%, and approximately 1–2% of these neonates develop early-onset infection [3].

Bacterial sepsis in newborns varies nationwide, even within the same country. Compared to developed countries, where the neonatal mortality rate is only 5 deaths per 1000 live births, the rate is 34 deaths per 1000 live births in developing countries, with most cases occuring in the first week of life [1].

Neonatal sepsis in developing nations is frequently caused by GBS and other bacteria, with significant morbidity and mortality. The morbidity and mortality from these infections varies based on the microorganisms and associated environmental, socioeconomic, and hygienic factors [4]. Nevertheless, nationwide studies concerning neonatal sepsis in Saudi Arabia are lacking.

Screening for GBS colonization and intrapartum antibiotics administration to pregnant women at high risk of GBS colonization has reportedly lowered the infection rates, thereby preventing early-onset GBS infection in their offspring [5].

The American College of Obstetricians and Gynecologists and the US Centers for Disease Control and Prevention recommend universal GBS screening. However, other bodies outside North America recommend risk-based GBS screening during pregnancy to guide the use of intrapartum antibiotic prophylaxis (IAP) [6].

Universal screening is described as screening all pregnant women at 35–37 weeks of gestation for GBS infection by vaginal and rectal culture testing. IAP is administered upon detecting a positive GBS culture. In risk-based screening, IAP is administered to mothers with risk factors for GBS, such as premature birth at or before 37 weeks of gestation, maternal fever of at least 38 °C, prolonged membrane rupture, and GBS bacteriuria during pregnancy [7].

No national guidelines exist for screening pregnant women for GBS (i.e., universal culture-based versus risk-based) in Saudi Arabia. Institutions have varying assessment levels [8]. Prenatal care in Saudi Arabia is provided at various health care facilities (i.e., primary health care centers, private sector, or governmental hospitals). Pregnant women can choose where to receive prenatal care and give birth. Physicians, such as family practitioners, obstetricians, and gynecologists, are the ones who perform these procedures.

This systemic review aimed to assess the prevalence of GBS colonization among pregnant women and the incidence of neonatal GBS infection, testing the hypothesis that the prevalence of GBS colonization among pregnant women and the incidence of neonatal GBS infection in the Saudi Arabian population are similar to the international rate.

## 2. Materials and Methods

### 2.1. Literature Search

A literature search was conducted for the following databases for relevant published studies: PubMed (1946 onwards), MEDLINE Ovid (1946 onwards), MEDLINE Ovid (In-Process and Other Non-Indexed Citations), PubMed (as a top-up to searches in MEDLINE), and Google Scholar. In addition, the search for different combinations of the following keywords was performed from January 1980 to December 2021: neonatal sepsis, neonatal group B infection, Group B *streptococci*, *Streptococcus agalactiae*, maternal colonization, intrapartum antibiotic prophylaxis, Saudi Arabia, guidelines, and recommendations.

### 2.2. Inclusion and Exclusion Criteria

The inclusion criteria of the studies were as follows: (1) the GBS colonization rate among pregnant women and (2) the incidence of neonatal GBS infections. All the studies were conducted in Saudi Arabia.

The GBS colonization of pregnant women (i.e., maternal colonization) is defined as a positive result of GBS bacterial culture from rectum, vagina or urine that was obtained during pregnancy.

Neonatal GBS infection is defined as a positive GBS culture from blood, urine, respiratory or cerebrospinal fluid associated with symptoms and signs of infection (e.g., fever, respiratory distress, poor feeding, seizure, increased respiratory or heart rate, hypotension, or hypoxia). The early-onset sepsis (EOS) is defined as infections that occur from birth through day 6 of life; while the late-onset sepsis (LOS) is defined as infections that occur from day 7 to day 28 of life.

The exclusion criteria of the studies were as follows: (1) studies that report neonatal infections but without data about GBS incidence, and (2) studies that include centers from outside Saudi Arabia.

### 2.3. Data Extraction

A standard data collection sheet was used to collect information from eligible studies. Studies were reviewed and data were abstracted by two reviewers: the author (AA) and Dr. Abdulsalam Alawfi (Acknowledged in the acknowledgment section) separately. Data collection included the first author, year of publication, sample size, and baseline characteristics of the enrolled sample. GBS colonization in pregnant women and neonatal GBS infection were the primary objectives of this study. The results of the included studies were also extracted.

### 2.4. Risk of Bias Assessment

The quality of the included observational studies was assessed using the Newcastle-Ottawa scale (NOS) tools for cohort, case–control, and cross-sectional studies (modified version). These tools are composed of multiple questions assessing any possible risk of bias concerning the selection process, comparability between groups, and outcome assessment.

The reporting of this systemic review was guided by the Preferred Reporting Items for Systematic Review and Meta-Analysis (PRISMA) Statement. However, it was not registered in any systemic reviews’ registry. 

## 3. Results

### 3.1. Literature Search

**Figure 1:**
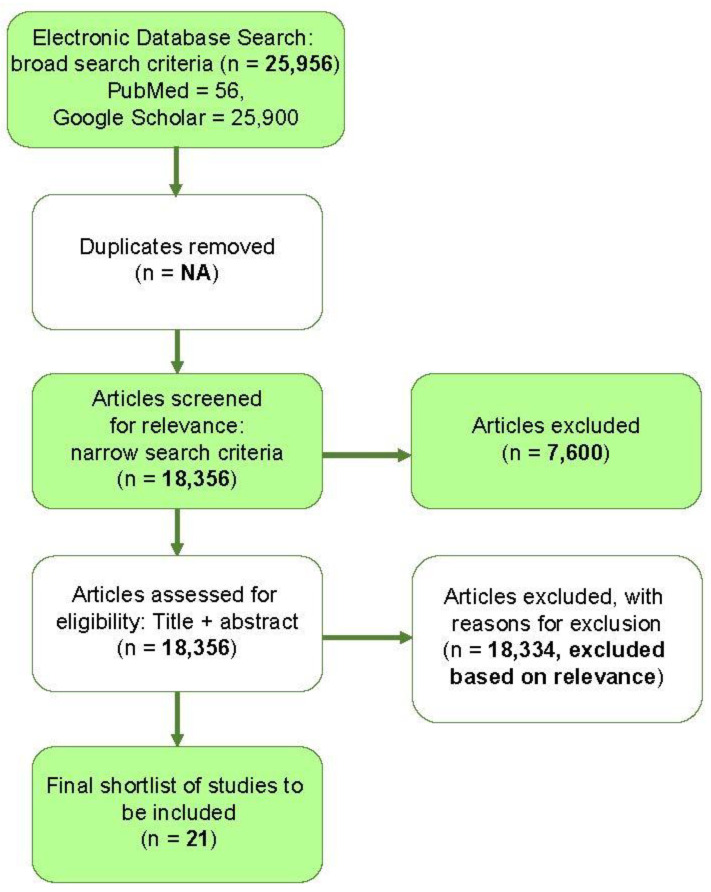
The flow diagram for study selection.

The initial search strategy resulted in 25,956 studies. After duplicate removal and screening, 21 articles were deemed reliable for review. The flow diagram for study selection is shown in Figure 1.

### 3.2. Characteristics of the Included Studies

This review included 21 studies conducted in Saudi Arabia between 1 January 1980 and 31 December 2021. Fifteen of the included studies assessed the prevalence of GBS colonization among pregnant women [8,9,10,11,12,13,14,15,16,17,18,19,20,21,22]; six studies assessed neonatal infection caused by GBS [23,24,25,26,27,28].

### 3.3. Risk of Bias Assessment

Per the NOS tools, the quality of the included studies was poor, except for one case–control study, which was of good quality [28], and one cross-sectional study, which was of fair quality [10]. The low quality of most of the included studies highlighted the lack of comparability (control group). The detailed result of bias assessment is shown in Supplementary Material Table S1.

### 3.4. Prevalence of GBS Colonization among Pregnant Women

The prevalence of GBS colonization varied considerably among the included studies, with Milyani and colleagues having the highest prevalence of colonization with 32.8% among pregnant women [10]. On the other hand, Ahmad and others reported the lowest prevalence of infection, with 2.1% of the study participants [11]. As in the studies by Milyani and colleagues, the studies by Zamzami et al., El-Kersh et al., Rabaan et al., El-Kersh et al., and Arain et al. had relatively high colonization among their participants, 31.6%, 27.4%, 25.09%, 23%, and 24%, respectively [10,12,13,14,15,16]. On the other hand, Hussain et al., Al-Sunaidi et al., and Uduman et al. demonstrated a relatively lower prevalence of colonization; 7.6%, 4.76%, and 9.2%, respectively [17,18,19].

Musleh et al., Khan et al., Mohamed et al., Al-Suleiman et al., and Khater et al. demonstrated an intermediate prevalence of colonization of GBS in their studies as 19%, 13.4%, 15%, 17.2%, and 16.1%, respectively [8,9,20,21,22].

Mohamed et al. and El-Kersh et al. described the distribution of GBS serotypes among the GBS colonized pregnant women [9,13]. In the study by Mohamed et al., serotype Ia was the most common (30%), while serotype II was the most found by El-Kersh et al. [9,13].

Characteristics and results of included studies of maternal GBS prevalence are shown in Table 1.

Eleven studies used the gold-standard post-enrichment subculture method for GBS screening and identification [9,10,12,13,14,15,16,18,20,21,22]. However, four studies used direct culture without enrichment media [8,11,17,19]. The prevalence of GBS colonization in these four studies were 19%, 2.1%, 7.6% and 9.2%, respectively [8,11,17,19].

Six studies have reported maternal risk factors as primary or secondary outcomes [8,10,14,15,16,21]. The advanced maternal age (>36 years) was the most common risk factor for GBS colonization [8,14,15,16,22]. In addition, Milyani et al. and El-Kersh et al. found that vaginal discharge was associated with a high colonization rate; 37% and 26%, respectively [10,15].

Four studies have reported the susceptibility of the GBS isolates [9,13,16,21]. GBS susceptibility to ampicillin, penicillin and vancomycin was 100% [9,13,16,21]. However, the susceptibility to clindamycin ranged from 85% to 99.8% [9,16]. A similar rate was observed for erythromycin susceptibility, which ranged from 83.3% to 99.6% [9,16].

### 3.5. Incidence of Neonatal GBS Sepsis

Dawodu and others conducted a study in 1997. They found that the incidence of sepsis in neonates during the study period was 4.9 per 1000 live births, of which 2 out of 61 neonates with sepsis were attributed to GBS infection [23]. They also declared that *Staphylococcus epidermidis* is the most isolated pathogen during EOS and LOS. On the contrary, Matary et al. found that the most common cause of EOS was GBS, representing 33.3% of neonates with EOS [25].

In a study by Almudeer and colleagues, EOS was found in 4.44 per 1000 live newborns. Among the neonatal specimens, *Escherichia coli* (29%) and GBS were the most detected pathogens (17%) [26].

Al Luhidan and others conducted their study for over 13 years and reported an incidence of GBS sepsis at 0.51 per 1000 live births; 69.1% had EOS [24]. GBS infection was identified in 23 of the 29,601 live births studied by Almuneef et al., resulting in an overall incidence rate of 0.8 per 1000 live births throughout the research period of 5 years [27].

Al Kadri and colleagues conducted a case–control study over ten years to determine the maternal and neonatal risk factors associated with early-onset GBS. They detected 99 cases of EOS attributed to GBS [28].

Four of the six included studies were from the central region [24,25,27,28], and three were from the same center [24,27,28]. Characteristics and results of included studies of neonatal GBS are shown in Table 2.

## 4. Discussion

GBS in the mother’s vagina, rectum, and urine increases the risk of neonatal infections. High mortality and catastrophic diseases, including sepsis and meningitis, are common among neonates infected with GBS in both developed and developing countries [7].

The results of the study by Khater and others were similar to those by Musleh and colleagues in 2018 [8,20], who observed that the colonization rate of Saudi women attending the King Fahd University Hospital during labor was 19% [8]. GBS positivity was 16.3% in a Makkah study conducted by Khan et al. in 2015 [21]. The colonization rate in the prior studies is higher than the overall colonization rate (12.7%) reported in a systemic review of 34 studies from 23 developing countries [29].

Higher prevalence rates were observed in studies conducted in Riyadh and Jeddah (27.6% and 31.6%, respectively) in Saudi Arabia [12,15]. Differences in the GBS colonization prevalence may be attributed to geographic location, age, parity, and socioeconomic status [30].

The colonization rates of GBS in the studies by Hussain et al., Al-Sunaidi et al., and Ahmad et al. were modest at 7.4%, 4.76%, and 2.1%, respectively [11,17,18]. However, Ahmad et al. included urine samples from pregnant women without a vaginal or rectal swab, which might explain the low GBS prevalence [11]. In 1985, research by Uduman and colleagues reported a colonization rate of 9.2% in mothers with GBS [19]. It is unclear whether the findings of Musleh et al. and Khater et al. represent a natural increase in the incidence of GBS colonization or, more likely, a shift in the sampling and culturing procedures [8,20].

A lower rate of GBS colonization in the studies by Ahmed et al., Hussain et al., and Uduman et al. could be explained by lack of using the standard post-enrichment subculture method of GBS screening and identification [11,17,19].

Maternal GBS colonization in the United States has steadily declined over the years and currently stands at 20–25% [31]. The prevalence of GBS colonization in Saudi Arabia could be further reduced by improving public awareness and the standardization of GBS screening practices.

Testing at 35–37 weeks of gestation could result in an erroneous impression of a decreased colonization rate. Therefore, timing is critical in this field. Based on a systematic review of the GBS screening process, screening at 35–37 weeks has positive and negative predictive values of 69% and 94%, respectively [32]. Different studies conducted in various regions of Saudi Arabia have reported varying rates of neonatal sepsis. According to Almudeer and others, 126 newborns were found to have EOS, with an overall rate of 4.44 per 1000 live births, with GBS representing 21 cases (17%). Moreover, 23% of the infants died within the first week of life [26].

Studies in Saudi Arabia have examined the EOS and LOS in tertiary health care facilities. Neonatal sepsis has been recorded in many newborns in King Fahad Medical City in Riyadh as per a retrospective study by Al-Matary et al. There were 298 newborn sepsis diagnoses between January 2011 and December 2015, with EOS accounting for 11.1% of the total. A third of newborns with EOS were found to have GBS, followed by *Escherichia coli* (27%) [25]. Almudeer et al. found a correlation between low birth weight, preterm delivery, and the risk of infection, such as EOS, in the study by Al-Matary and others [25,26].

Only two [24,27] of the six included studies in neonates have reported the incidence of neonatal GBS infection per 1000 live -births, which is the standard reporting method of neonatal GBS infections [3]. In these two studies, the overall incidence of neonatal GBS was 0.51 and 0.8 per 1000 live births, respectively [24,27]. However, in the study by Al Luhidan and others, the incidence increased in 2015 and 2016 to 1.6 and 1.8, respectively. This increase coincided with the discontinuation of universal GBS screening at the study center [24].

The incidence of neonatal GBS infection in these studies is consistent with another prospective research on EOS for two years from five hospitals in three Arab gulf countries including Saudi Arabia, The United Arab Emirates, and Kuwait [33]. GBS infections were the most common cause of EOS in their samples, accounting for over 60% of the cases, followed by *Escherichia coli* infections. The overall incidence of EOS due to GBS was 0.90 per 1000 live births, ranging from 0.6 to 1.4 per 1000 live births [33]. However, the incidence rate might be higher in the preceding study as the authors shortened the duration of EOS to 72 h rather than the standard definition of 6 days [33].

The data regarding the incidence of neonatal GBS infections from the studies included in this review are not conclusive due to the lack of nationwide data [24,27]. However, this review showed that the incidence of neonatal GBS infection in Saudi Arabia is higher than the overall international incidence as per a systemic review and meta-analysis by Madrid et al., which included 135 studies worldwide. The pooled incidence of invasive GBS disease in infants was 0.49 per 1000 live births (95% confidence interval: 0.43–0.56) [34].

None of the included studies reported the serotype in the neonates. However, two studies reported the serotypes of GBS in pregnant women [9,13]. The serotypes Ia and II were the most common [9,13]. This was inconsistent with most common serotype worldwide which is serotype III (61.5%) [34].

The susceptibility of GBS to ampicillin and penicillin was 100% in all studies that reported the susceptibility results [9,13,16,21]. In addition, the susceptibility of clindamycin and erythromycin remained high with rate ranging from 84% to 99.6% [9,16]. The rate of clindamycin susceptibility was higher than the rate reported from the United States and China which showed a susceptibility rate of 70.8% and 10%, respectively [35,36]. A similar lower rate of erythromycin susceptibility was reported in these two studies [35,36].

This study has the strength of being the first systemic review conducted in Saudi Arabia to assess the prevalence of GBS among pregnant women and the incidence of GBS sepsis in neonates. This review included a broad specified search over a considerable period, from 1980 until the end of 2021. It included a significant number of studies regarding maternal GBS colonization from different regions. However, limitations include the small number of studies regarding neonatal GBS incidence and outcomes with heterogenicity of the reporting methods Therefore, further original studies are required to fill the knowledge gaps regarding the accurate incidence and burden of GBS infection among neonates in Saudi Arabia. In addition, further studies are required to determine the risk factors resulting in the high prevalence of GBS colonization among Saudi Arabian women.

## 5. Conclusions

Studies that assess maternal GBS colonization in Saudi Arabia are limited to single-center studies. However, the prevalence of maternal GBS colonization is higher than the average rate of other countries. On the other hand, the data regarding neonatal GBS infections are limited and inconclusive, with only two studies reporting the incidence of neonatal GBS infection as the primary outcome. Therefore, nationwide studies and registries are warranted to assess the burden of neonatal GBS infection. These studies would guide the appropriate GBS screening strategies during pregnancy for application in a national public health program.

## Figures and Tables

**Table 1:** Characteristics and results of GBS prevalence studies among pregnant women.

Study ID	Region	Design	Outcomes	Sample Size	Collection Site	Colonization/Infection Rate N (%)
Musleh et al., 2018 [8]	Eastern	Cross sectional	Prevalence of GBS ^1^ colonization	457	Vaginal Rectal Urine	87 (19)
Mohamed et al., 2020 [9]	Western	Cross sectional	- Prevalence of GBS colonization - Serotypes - Susceptibility pattern	400	Vaginal Rectal	60 (15) Serotype Ia (30%)
Milyani et al., 2011 [10]	Western	Cross sectional	Prevalence of GBS colonization	119	Vaginal Rectal	39 (32.8)
Ahmed et al., 2015 [11]	Central	Cross sectional	Prevalence of GBS bacteriuria at first and second trimesters	3863	Urine	82 (2.1)
Zamzami et al., 2011 [12]	Western	Cross sectional	Prevalence of GBS colonization at 33 weeks gestation and birth	326	Vaginal Rectal	103 (31)
El-Kersh et al., 2012 [13]	Central	Cross sectional	- Comparing antigen detection method with standard culture method - Serotypes -Susceptibility pattern	217	Vaginal	50 (23) serotype II (42%)
Rabaan et al., 2017 [14]	Eastern	Cross sectional	Comparing PCR detection method with standard culture method	554	Vaginal Rectal	139 (25)
El-Kersh et al., 2002 [15]	Central	Cross sectional	- Prevalence of GBS colonization - Assessing specimen types and techniques for GBS detection	217	Vaginal Rectal	66 (27.6)
Arain et al., 2015 [16]	Western	Cross sectional	- Prevalence of GBS colonization - Susceptibility pattern	2632	Vaginal Rectal	632 (24)
Hussain et al., 2015 [17]	Central	Retrospective	Prevalence of GBS colonization	3253	Vaginal Rectal	246 (7.6)
Al-Sunaidi et al., 2011 [18]	Southern	Cross sectional	Prevalence of GBS colonization	105	Vaginal	5 (4.8)
Uduman et al., 1985 [19]	Eastern	Cross sectional	Prevalence of GBS colonization	260	Vaginal	24 (9.2)
Khater et al., 2021 [20]	Central	Cross sectional	Prevalence of GBS colonization	540	Vaginal Rectal	87 (16.1)
Khan et al., 2015 [21]	Western	Cross sectional	Prevalence of GBS colonization	1328	Vaginal	178 (13.4)
Al-Suleiman et al. [22]	Eastern	Cross sectional	Prevalence of GBS colonization	1939	Vaginal Rectal Urethral	334 (17.2)

^1^ GBS: Group B *Streptococcus;* PCR: polymerase chain reaction.

**Table 2:** Characteristics and results of neonatal GBS sepsis incidence and outcome studies.

Study ID	Region	Design	Outcomes	Overall Incidence of GBS Sepsis N (per 1000 Live Births)	Incidence of EOGBS N (per 1000 Live Births)	Incidence of LOGBS N (per 1000 Live Births)	CFR N (%) *	Screening Strategy: Universal (U) Risk Based (R) NR
Dawodu et al., 1997 [23]	Eastern	Cross sectional	Incidence of neonatal sepsis (overall)	2 (0.18)	2 (0.18) *	0	1 (50)	NR
Al Luhidan et al., 2019 [24]	Central	Retrospective cohort	Incidence of GBS sepsis	55 (0.51)	38 (0.34)	17 (0.15)	2 (3.6)	Both U and R
Al-Matary et al., 2019 [25]	Central	Retrospective cohort	Incidence of neonatal sepsis (overall)	NR	33% of all EOS	1.9% of all LOS	NR	NR
Almudeer et al., 2020 [26]	Southern	Retrospective cohort	Incidence of EOS (overall)	21 (0.74)	21 (0.74) *	NA	NR	NR
Almuneef et al., 2000 [27]	Central	Retrospective cohort	Incidence of GBS sepsis	23 (0.8)	19 (0.6)	4 (0.2)	2 (9)	NR
Al-Kadri et al., 2013 [28]	Central	Case–control	Neonatal and maternal risk factors for EOGBS	NR	NR	NR	NR	R

* Calculated from the studies’ results. GBS: Group B *Streptococcus*; EOGBS: early-onset Group B *Streptococcus*; LOGBS: late-onset Group B *Streptococcus*; CFR: case fatality rate; NR: not reported; NA: not applicable.

## Data Availability

Not applicable.

## Supplementary Materials

The following supporting information can be downloaded at: https://www.mdpi.com/article/10.3390/pathogens11091029/s1, Table S1: Newcastle-Ottawa scale (NOS) tools.
